# Assessing direct and indirect effects of pediatric influenza vaccination in Germany by individual-based simulations

**DOI:** 10.1080/21645515.2019.1682843

**Published:** 2019-12-06

**Authors:** Ruprecht Schmidt-Ott, Daniel Molnar, Anastassia Anastassopoulou, Emad Yanni, Claudia Krumm, Rafik Bekkat-Berkani, Gaël Dos Santos, Philipp Henneke, Markus Knuf, Markus Schwehm, Martin Eichner

**Affiliations:** a GSK, Wavre, Belgium; b GSK, Munich, Germany; c GSK, Rockville, MD, USA; d GSK, Philadelphia, PA, USA; e Center for Chronic Immunodeficiency and Center for Pediatrics and Adolescent Medicine, Medical Center, Faculty of Medicine, University of Freiburg, Freiburg, Germany; f Helios Dr Horst Schmidt Kliniken Wiesbaden, Wiesbaden, Germany; g ExploSYS GmbH, Leinfelden-Echterdingen, Germany; h Epimos GmbH, Dusslingen, Germany; i University of Tübingen, Tübingen, Germany

**Keywords:** Influenza, vaccination, pediatric, simulation, mathematical model

## Abstract

Children have a high burden of influenza and play a central role in spreading influenza. Routinely vaccinating children against influenza may, thus, not only reduce their disease burden, but also that of the general population, including the elderly who frequently suffer severe complications. Using the published individual-based tool 4Flu, we simulated how pediatric vaccination would change infection incidence in Germany. Transmission of four influenza strains was simulated in 100,000 individuals with German demography and contact structure. After initialization with the recorded trivalent influenza vaccination coverage for 20 years (1997–2016), all vaccinations were switched to quadrivalent influenza vaccine (QIV). Scenarios where vaccination coverage of children (0.5-17-year-old) was increased from the current value (4.3%) to a maximum of 10-60% were compared to baseline with unchanged coverage, averaging results of 1,000 pairs of simulations over a 20-year evaluation period (2017–2036). Pediatric vaccination coverage of 10-60% annually prevented 218–1,732 (6.3–50.5%) infections in children, 204–1,961 (2.9–28.2%) in young adults and 95–868 (3.1–28.9%) in the elderly in a population of 100,000 inhabitants; overall, 34.1% of infections in the total population (3.7 million infections per year in Germany) can be prevented if 60% of all children are vaccinated annually. 4.4–4.6 vaccinations were needed to prevent one infection among children; 1.7–1.8 were needed to prevent one in the population. Enhanced pediatric vaccination prevents many infections in children and even more in young adults and the elderly.

## Introduction

Epidemiological evidence from Finland and the US consistently indicates high rates of influenza infection in young children, with typically 15–30% of children acquiring symptomatic infection each year.^1^ Recent studies have documented that infants and young children without underlying medical conditions are hospitalized for influenza-attributable illnesses at rates that are similar to those of adults with high-risk conditions.^2^ Antigen-matched influenza vaccines are efficacious in preventing influenza.^3–5^ Two recent large randomized controlled studies with quadrivalent inactivated influenza vaccines (QIV) have confirmed this also for children aged 6 months to 3 years.^6,7^ The World Health Organization (WHO) includes children aged 6 to 59 months in their recommendations on routine influenza vaccination, in addition to other vulnerable risk groups, i.e. pregnant women, the elderly, individuals with specific chronic medical conditions, and people with increased risk of exposure.^8^ This risk-based vaccination strategy is also reflected in the European Council’s recommendation on seasonal influenza vaccination, albeit that recommendation from 2009 does not mention young children as a particular risk group.^9,10^

Consequently, only few European countries include healthy children in their target groups for routine vaccination, e.g. children aged 2 to 16 years in the UK or infants 6 months to 2 years in Finland. Most European countries, among them Germany, only recommend vaccinating children who have underlying chronic diseases.

Children are not only susceptible for influenza infection and its complications, but they also play an important role in spreading influenza infections in the community. Thus, targeting children in vaccination campaigns may not only reduce their individual influenza burden, but also that of non-vaccinated individuals. Observations from different countries support this hypothesis. In the US, vaccination of 25% of children (2–18 years) was associated with a reduction of the physician consultation frequency for respiratory illness by up to 18% for adults ≥ 35 years of age.^11^ In Canada, vaccination of 83% of children under 16 years of age was accompanied by a 61% reduction of influenza infection of unvaccinated individuals.^12^ Data from Japan show that vaccination of school-age children indirectly reduced influenza mortality in the elderly.^13^ More recently, vaccination of primary school children in the UK reduced influenza-related medical outcomes in adults.^14^

Mathematical modeling has helped to quantify direct and indirect effects of influenza vaccination on a population level. Transmission models have been used in different settings to estimate the overall and age-specific number of infections that can be prevented by vaccinating children.^15^ In the present study, we use the dynamic individual-based simulation tool 4Flu (https://www.4flu.net^16,17^) to examine how routine childhood influenza vaccination may change the annual infection incidence in Germany.

## Material and methods

The employed dynamic simulation tool 4Flu has already been described in detail^16-18^ and is freely available on the web (https://www.4flu.net). A comprehensive list of the parameters used in the model, together with references from which sources these parameters were derived, is also provided in the online supporting material (Table A1). 4Flu is an individual-based tool which simulates the independent spread of the four currently circulating influenza viruses A(H1N1), A(H3N2), B/Yamagata and B/Victoria in a population with dynamically changing demography and contact patterns.

### Simulated population

At the beginning of the evaluation period, the simulated population contains 100,000 individuals. Simulated births and deaths cause the population to change dynamically, mirroring German demographic observations and predictions.^19^ As the vaccination coverage differs for individuals with underlying medical conditions (“at risk” individuals), we also consider the risk status of individuals (for more details on model parameters, see Online Supporting Material Table A1). Individuals are interconnected in a contact network which is based on the German POLYMOD study.^20^ As the age-distribution of the population changes during the simulation period, the overall contact structure must also dynamically adapt to these demographic changes. Existing connections between individuals are continuously removed and new contacts are formed throughout the simulation to keep the individuals’ contacts aligned to the Germany POLYMOD matrix despite of their aging and despite of the demographic changes of the population (details of how this is achieved are given in^17^). For rendering realistic age-dependent immunity patterns in the simulated population, the simulation starts 20 years in the past (i.e. on 1 September 1997). During that period, individuals are born, age and die; they may be infected with influenza and may be vaccinated with trivalent influenza vaccine (TIV).

### Natural history

As infection transmission can die out in the relatively small simulated population, infections from the “outside” are introduced at random time points. Infective individuals pass on the infection to some of their contacts. Children have been found to shed influenza virus for a longer period of time than adults and, thus, can infect more of their contacts.^21-23^ Influenza infections follow a strong seasonal pattern with typical peaks in January or February.^24^ To account for seasonal fluctuations, the average transmission probability per contact per day is multiplied with a factor which depends on calendar time and which is assumed to reach a maximum on 21st December.^25^ Due to genetic changes of the virus, naturally acquired immunity to circulating strains continuously declines over time, but it can later be boosted by infections or vaccinations. In randomly chosen years, new drift variants of any one of the four influenza types are introduced. These are only partially similar to the previously circulating variant with respect to their immunologic fingerprint and, therefore, become the new dominant circulating strain. The introduction of drift variants (which takes place on average twice in 7 years for A(H3N2) or once in 7 years for the other influenza strains) is associated with an additional loss of immunity which reduces the average duration of immunity to 4.5 years for A(H3N2) and to 6 years for the other lineages,^16^ respectively. The two B lineages share some cross-immunity, i.e. infection with one influenza B virus lineage can induce or boost immunity against the other.

### Vaccination

In Germany, vaccinations are performed annually in October and November, in line with current recommendations.^26^ Vaccination coverage depends on the age and risk status of the individuals (Online Supporting Material Table A2). Vaccinees of the previous season have a higher probability to be re-vaccinated than previously unvaccinated individuals. The vaccine efficacy depends on the age of the vaccinee.^3-5^ Following official national guidance^27^ and the summary of product characteristics of inactivated influenza vaccines currently available in Germany, children below 9 years who previously were not vaccinated against influenza receive two vaccinations shortly after each other in our simulations; the same vaccine efficacy is assumed for this “double-vaccination” as for normal single vaccinations. In some of the years when a new drift variant occurs, the vaccine efficacy is reduced against the new variant because of a vaccine strain mismatch. Vaccination-derived immunity lasts until the end of the simulation year in which the vaccination occurs (i.e. until 31 August),^28^ but it can be boosted by infections. Vaccinations can also boost infection-derived immunity.

### Model calibration

The transmission probability was calibrated such that in 2006/07, the median infection incidence in young adults (obtained by 10,000 simulations) reached the reported value of 10.6%.^29^ This was obtained by setting the average transmission probability to 3.05% per contact per day.^16,29^ Applying the raw data from the original publication^29^ (78 infections in 736 adults), we calculated that the 95% confidence interval (CI) for the incidence of infection is (8.5%, 13.07%) per year. Using the lower and the upper limit of this CI as alternative calibration targets lead to average transmission probabilities per contact per day of 2.8 and 3.3%, respectively. We used these values in one univariate sensitivity analysis.

### Evaluation of results

On 1 September 2017, the 20-year evaluation period starts during which only QIV is used. In this period, simulations are split into two simulation branches which run in parallel (these simulation branches only differ in the vaccination strategy, whereas all demographic events like births and deaths and changes in the contact network are identical). In branch 1, the same vaccination coverage is used as in the initialization period; in branch 2, the vaccination coverage of a selected group of children is gradually increased within four years. In each branch of the simulation, the numbers of infected individuals are recorded daily, taking into account the age of the infected individuals and their risk status. Finally, the number needed to vaccinate (NNV) in order to prevent one infection is calculated by dividing the number of additional vaccinations by the number of additionally prevented infections.

### Simulation studies

In the baseline comparisons, the vaccination coverage of children in branch 2 is increased to 40% (irrespective of their risk status) either (a) for pre-school children (0.5–4 years) or (b) for all children (0.5–17 years), or (c) it is increased to 90% for at-risk children only (0.5–17 years), without changing the vaccination coverage of the other age groups (1,000 pairs of simulations for each target age group). In scenario analyses, we use vaccination coverage values of 10 to 60% instead of 40% for the 0.5 to 4 and for the 0.5 to 17 years age-group (1,000 pairs of simulations for each combination of target age group and vaccination coverage). The 25th and 75th percentiles (Q1 and Q3 respectively) of estimated numbers and percentages of prevented infections are provided in the Online Supporting Material (Table A3 – A6). To determine the influence of selected parameters on the results, univariate sensitivity analyses are performed in which one parameter at a time is either set to an assumed minimum or an assumed maximum value: (1) The loss rate of naturally acquired immunity is either set to half or twice the baseline value (4.56 vs. 18.26 years). (2) For the individual transmission probability per contact per day, the lower and upper limits of the 95% CI (obtained by model calibration as explained above) are used (2.8% vs. 3.3%). (3) The lower and upper limits of the 95% CIs of the age-dependent (0–2 years/3–8 years/9–15 years) vaccine efficacy values are used (42.8%/39.1%/55.0% vs. 56.0%/67.3%/78.0%).

### Age-specific transmission patterns

To quantify age-specific transmission patterns, we randomly picked one simulation with unchanged vaccination coverage (i.e. branch 1) and recorded throughout the 20-year evaluation period for every infected individual how many children (0–17 years), young adults (18–59 years) and the elderly (60+ years) were infected by these individuals.

### Comparison of simulation results with seroprevalence data

We performed 3,000 simulations with 100,000 individuals in which we recorded for each individual whether an Influenza A infection (A(H1N1) or A(H3N2)) or an Influenza B infection (B/Victoria or B/Yamagata) or any influenza infection had occurred during the season 2008/09 to 2010/11. These simulations were run with the vaccination coverage of these seasons, using inactivated trivalent vaccine (TIV) which contained the recorded B lineage. Simulation results were compared to published sero-prevalence data which reported the fraction sero-positive to A (s_A) and the fraction sero-positive to B (s_B). The fraction sero-positive to any influenza infection (s_AB) was not reported in the publications; we calculated it by assuming that Influenza A is transmitted independently from Influenza B as s_AB = 1-(1-s_A)*(1-s_B).

## Results

**Figure 1.**
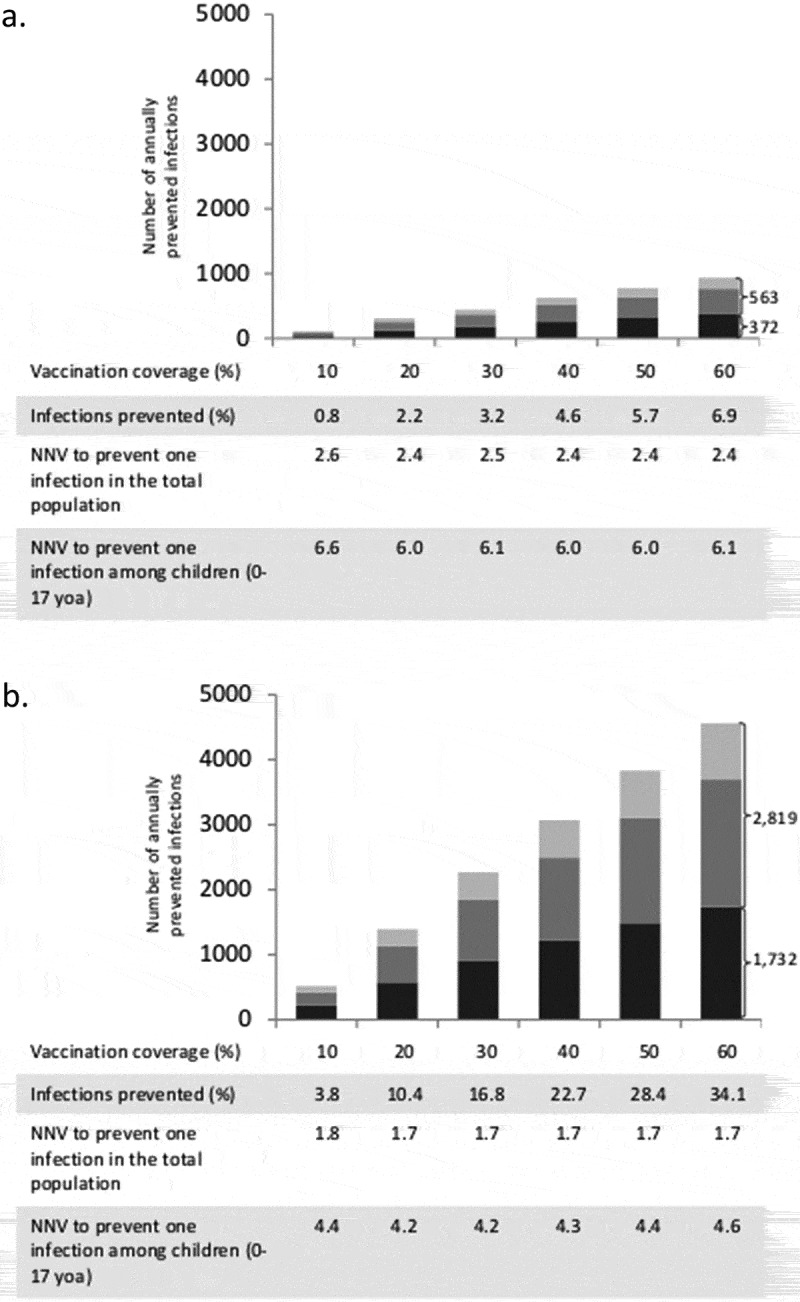
Increased QIV vaccination of children from 6 months to 4 years of age (a) or 6 months to 17 years of age (b). Average annual number of influenza infections prevented among children (black; 0–17 years), young adults (dark gray; 18–59 years) and elderly (light gray; 60+ years) compared to QIV vaccination at baseline coverage. Infections prevented (%) show the overall reduction of infection in the population. Averages of 1,000 pairs of simulations for each combination of target age-group and coverage; population size 100,000; evaluation period 20 years. NNV = number needed to vaccinate; yoa = years of age.

**Figure 2.**
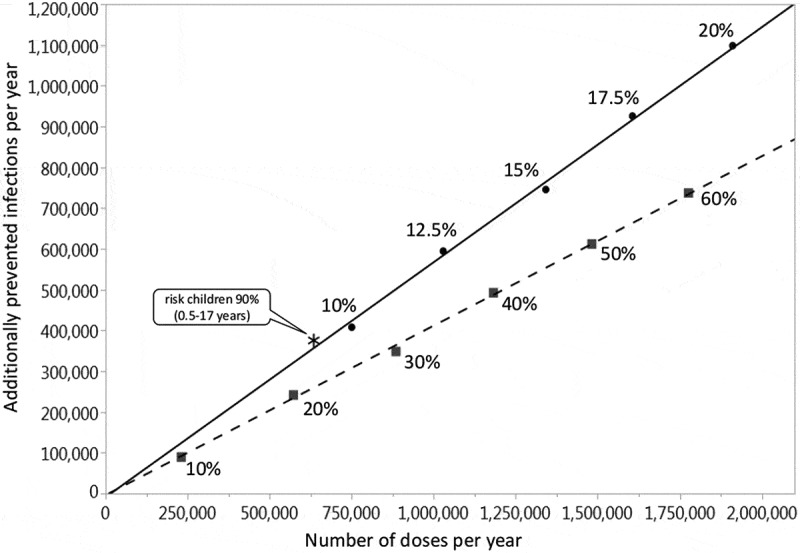
Prevented influenza infections in the German population by annually applying a number of QIV doses to pre-school children (0.5–4 years, squares with dashed regression line) or to all children (0.5–17 years, circles with full regression line) or to at-risk children (0.5–17 years; asterisk). The percentages next to the dots indicate what annual coverage of the respective group of children can be reached with the given number of doses. The origin of the curves indicates that no additional vaccinations are performed, i.e. the pediatric vaccination coverage in both simulation branches is equal to 4.1% (i.e. 7.7% for risk children, 3.8% for others). The figure also takes into account that children below 9 years of age are given two doses when they are vaccinated for the first time in their life. Averages of 1,000 pairs of simulations for each dot, using an evaluation period of 20 years; results are based on a simulated population size of 100,000 individuals and were extrapolated to the total population of Germany.

**Figure 3.**
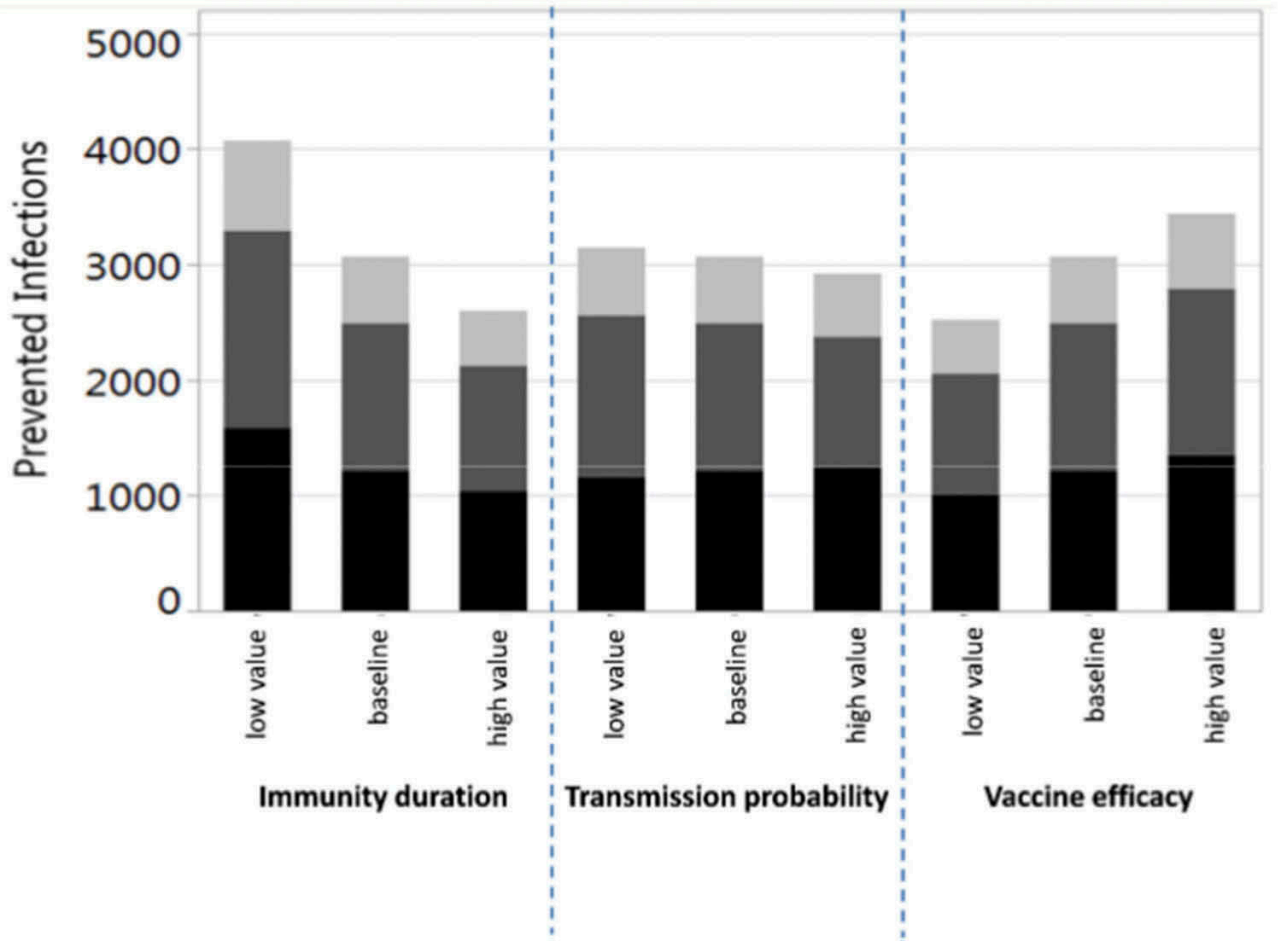
Univariate sensitivity analyses for the number of prevented infections when vaccinating 40% of children from 6 months to 17 years of age with QIV in a population of 100,000 individuals with German demography (annually prevented infections; black: children 0–17 years, dark gray: young adults 18–59 years, light gray: elderly 60+ years; averages of 1,000 pairs of simulations for each parameter setting, using an evaluation period of 20 years).

The baseline simulation results are summarized in Table 1. Vaccination of 40% of all children from 0.5 to 17 years prevents on average 35.2% (Q1-Q3: 32.9%-37.4%) of all infections among children and 22.7% (Q1-Q3: 20.3%-25.2%) in the total population (in the German population of 80.7 million, this corresponds to 2.5 million infections per year). It is noteworthy that more infections are prevented in adults (i.e. in the age groups where no additional vaccinations are performed) than in the vaccination target group of children. If 40% of all children are vaccinated, on average 4.3 vaccinations are needed to prevent one infection in children, whereas only 1.7 vaccinations are needed to prevent one infection in the total population. If, instead, 40% of only the pre-school children (0.5–4 years) are vaccinated, 7.2% (Q1-Q3: 5.4%-9.1%) of infections are prevented in children and 4.6% (Q1-Q3: 2.4%-6.7%) in the total population (corresponding to 0.5 million infections per year in Germany). If additional pediatric vaccinations are restricted to at-risk children (0.5–17 years; current recommendation), only 3.5% (Q1-Q3: 1.4%-5.6%) of infections in the total population can be prevented even at a vaccination coverage rate of 90%. The vaccination effects, which are obtained when varying the maximum vaccination coverage from 10% to 60% for all children (0.5–17 years), are depicted in Figure 1. Up to 34.1% (Q1-Q3: 31.4%-36.7%) of infections can be prevented in the total population if 60% of all children are vaccinated annually (this corresponds to 3.7 million infections per year in Germany). For vaccination scenarios targeting all children, the NNV ranges from 1.7 to 1.8 to prevent one infection in the total population. If only pre-school children from 0.5 to 4 years are vaccinated, the vaccination effects become much smaller: 0.8 to 6.9% of the infections in the total population can be prevented, and the NNV ranges from 2.4 to 2.6 (Figure 1; further numerical results are given in Tables A3 and A4 of the Online Supporting Material). Irrespective of the target age-group and the vaccination coverage, indirect vaccination effects in adults are consistently higher than the effects in children in terms of number of influenza infections prevented. For a better comparison between the scenarios targeting all children or only pre-school children, results are displayed in Figure 2 by the number of vaccine doses administered. For a given number of vaccine doses distributed over all children (0.5–17 years), more infections can be prevented than if these are distributed only to young children (0.5–4 years). In sensitivity analyses, we examined the robustness of our results by varying selected parameter values. Varying the loss rate of naturally acquired immunity had the biggest impact on the results, followed by the vaccine efficacy, whereas the contagiousness of influenza had the lowest impact (Figure 3; for more details, see Online Supporting material).

**Table 1.** Baseline results of vaccinating an increased percentage of children with QIV in a population of initially 100,000 individuals with German demography (annually prevented infections; averages of 1,000 pairs of simulations for each scenario, using an evaluation period of 20 years each).

Baseline QIV vaccination coverage is increased in four years to …	Annual number of infections prevented in				Number needed to vaccinate (NNV) to prevent one infection	
	children 0–17^#^	adults 18–59^#^	elderly 60+^#^	all ages^#^	among children	in the population
40% for pre-school children (0.5–4 years)	251 (7.2%)	261 (3.7%)	112 (3.6%)	624 (4.6%)	6.0	2.4
40% for all children (0.5–17 years)	1,216 (35.2%)	1,277 (18.2%)	574 (18.9%)	3,066 (22.7%)	4.3	1.7
90% for at risk children (0.5–17 years)	201 (5.8%)	189 (2.7%)	86 (2.8%)	476 (3.5%)	4.0	1.7

^#^Average annual population size during the evaluation period: Children 0–17 years: 14,565; Adults 18–59 years: 49,169; Elderly 60+ years: 34,010; All ages: 97,744 (deviating from the initial value of 100,000 according to the predicted demographic development of Germany).

The comparison of simulated sero-prevalence results for 2009–11 with observations from Germany and The Netherlands are shown in Figure A2 in the Online Supporting Material. Comparing our simulation results for this time period with observations made in a prospective study in Finland and assuming that 66.9% of all infections lead to clinical symptoms,^30^ we obtained a median annual incidence of clinical influenza infections of 14.2% in children below 14 years of age, which was slightly below the observed value of 16.7%.^2^

## Discussion

**Figure 4.**
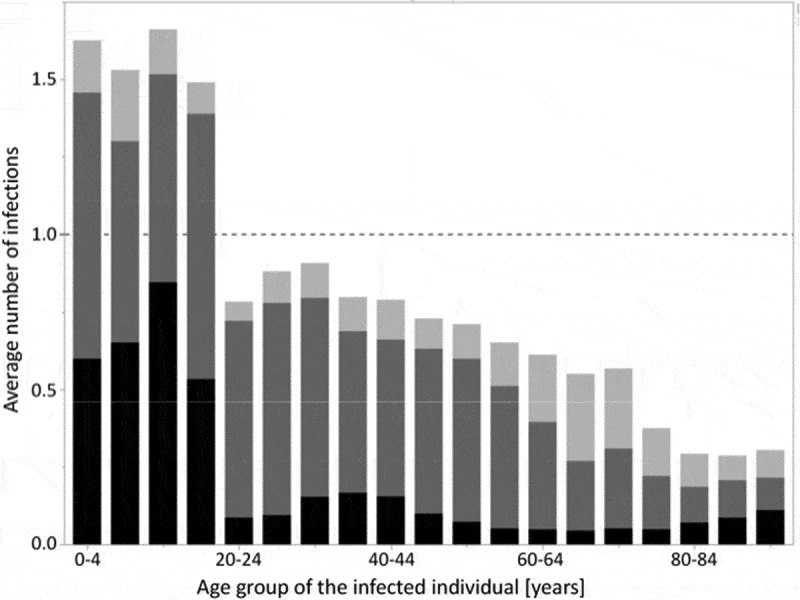
Average numbers of secondary infections per infected individual in 5-year age groups (starting with 0–4 years, 5–9 years, etc.). During the 20-year evaluation period of branch 1 (QIV vaccination with unchanged vaccination coverage) of a randomly picked simulation with 100,000 individuals, the numbers of secondary infections were recorded for all infected individuals. These numbers were then averaged for each 5-year age group (black: children 0–17 years, dark gray: young adults 18–59 years, light gray: elderly 60+ years).

**Figure 5.**
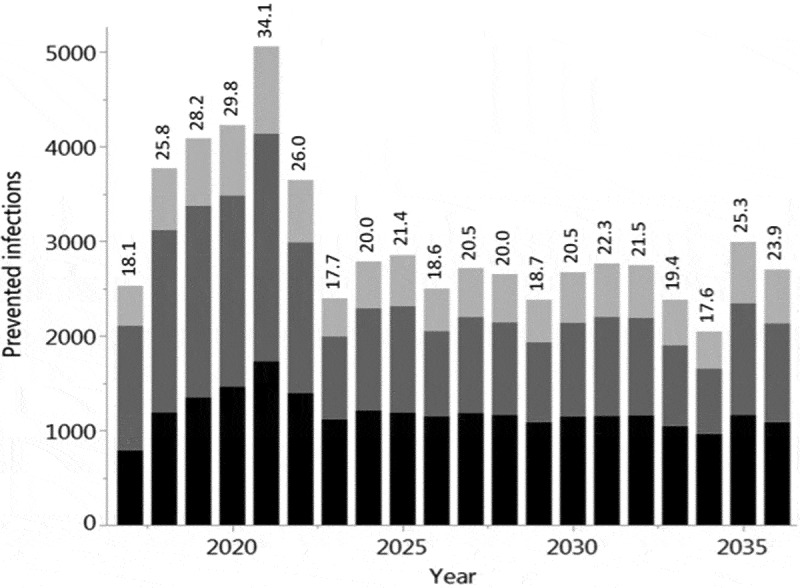
Time course of the annual number of prevented influenza infections during the 20-year evaluation period. Comparison between QIV vaccination at baseline coverage and extended QIV vaccination of up to 40% (reached after four years, using a linear increase) of vaccination of children from 0.5 to 17 years of age. The results show averages of 1,000 stochastic simulations with a population of 100,000 individuals (black: children 0–17 years, dark gray: young adults 18–59 years, light gray: elderly 60+ years). The numbers above the bars show the percentages of prevented infections in all age categories. Averaged over all 20 years, 3,066 infections (22.71%) are prevented annually.

In this work, we have analyzed the impact of changes in the current influenza vaccination practice in Germany on the incidence of influenza infections in different age groups using an individual-based dynamic transmission model. We took into account a recent survey of the Robert Koch Institute in Germany on the parents’ willingness to have their children vaccinated against seasonal influenza if these vaccinations were officially recommended and provided free of charge.^31^ Accordingly, we assumed vaccination coverage of 40% in children in our baseline scenarios. Analyses of social mixing patterns in several European countries based on the POLYMOD study^20^ showed that children have higher contact rates than adults and that they share more than half of their contacts with adults, whereas adults have most of their contacts among themselves.^17^ Looking at which individuals produce on average more than one secondary infection per infected individual allows determining which age-groups propagate the spread of influenza. In our simulations, infected children produce on average more than one secondary infection whereas infected adults infect less than one other person (Figure 4). This strongly supports the view that children are important drivers of influenza transmission in the community.^32-34^ These findings also suggest that increasing vaccination rates in children is an effective measure to reduce the influenza burden of the population. Our modeling results confirm that routinely vaccinating 0.5 to 17 years old children significantly reduces the incidence of influenza in both the target age groups and in the remaining population. According to the model, a vaccination rate of 40% in these children reduces the infection incidence of the entire German population by approximately one fifth (22.7%), preventing on average 2.5 million infections every year. For all vaccination coverage rates from 10 to 60%, the number of indirectly prevented infections in adults invariably exceeds the number of prevented infections in the target pediatric age group, resulting in low numbers needed to vaccinate (NNV≤1.8) to prevent one infection (Figure 1). These pronounced herd effects may be explained by the high number of daily contacts of children – not only among themselves, but also with adults^17^– and their long duration of infectiousness. Our modeling results are compatible with clinical studies and real world findings on indirect effects caused by vaccinating children, but they are more conservative than these findings: (1) In the US, vaccination of 25% of children (2–18 years) was associated with a significantly reduced physician consultation rate for respiratory illness in adults.^11^ Our simulations with a vaccine uptake of 25% in children (0.5–17 years) prevent on average only 11% of infections in young adults and the elderly (see Table A3 in the Online Supporting Material). (2) In a cluster randomized study performed in Hutterite communities in Canada, vaccination of 83% of children under 16 years of age was linked to a 61% reduction in influenza infections in all unvaccinated individuals.^12^ Again, our simulation results are more conservative. The extrapolation of our results to a pediatric coverage rate of 83% indicates that the indirect reduction for young adults and the elderly should be around 40% (Table A3 in the Online Supporting Materials). (3) During a recent pediatric pilot vaccination programme in England, a 59% reduction in medical consultations for influenza-like illness (ILI) among adults was observed after vaccinating 56.8% of primary school children (4–11 years).^14^ Our findings are more conservative than these data. In our simulations, vaccination coverage of 55% in children of 6 months to 17 years indirectly reduces influenza infections by 26% in young adults and the elderly. The differences between these published findings and our modeling results may be due partly to differences between the studied populations. A more important contributing factor may be that our simulation output is averaged over 20 years whereas most real-world findings cover only a small time period shortly following the change in vaccination strategy. We have chosen to use such a long time period because we wanted to avoid over-optimistic results, which invariably occur shortly after the rise of the vaccination coverage. The transient period of over-optimistic results which follow increasing the vaccination coverage has been called “honeymoon period”;^35^ it is caused by a combination of inherent natural immunity, which was acquired when the infection incidence was high, i.e. before vaccinations were increased, and the newly acquired immunity which is due to increased vaccination. In our simulations, pediatric vaccination is increased for four years, and the year with the highest number of prevented infections is reached in year five (Figure 5). After some years with increased vaccination coverage, new age-dependent transmission patterns establish and, thus, diminish indirect vaccine effects. This is in line with results of mathematical models based on differential equations, which made much more simplified assumptions when modeling immunity dynamics and more optimistic assumptions when modeling vaccination effects.^15,25,35,36^ A recent publication by Weidemann *et al*. used a deterministic transmission model to reconstruct the epidemiology of seasonal influenza in Germany by imposing that the strain-specific influenza immunity varied widely in the different years.^28^ They found that an increased vaccination rate in children leads to strong indirect effects when applying the vaccination coverage obtained from health insurance claims data to the other age groups. Their approach differs from ours in several aspects. Most importantly, they do not assume that the immunity at the end of a season depends on the number of infections which occurred during that season; instead of making this assumption, they estimate the strain-specific immunity pattern at the start of each simulation year such that their simulation results for the following season resemble observations (cf. their Figure 2). It may, thus, be surprising that they reported a 22.9% overall reduction in influenza-attributable medically attended acute respiratory infections when increasing the vaccination of 2–17 years of aged children to 40%, which is nearly identical to the 22.7% overall reduction reported here for the same coverage of 0.5–17 years of aged children (Table 1).

Increasing vaccination only in pre-school children aged 0.5 to 4 years would also lead to indirect effects in adults and the elderly which exceed the effects in the target age group; yet these indirect effects are much smaller than those which can be achieved by including older children as well (Figure 1). This is largely due to the higher number of contacts of older children and juveniles.^20^ Approximately the same numbers of vaccine doses are needed to vaccinate 45% of pre-school children or 15% of children up to 17 years (Figure 2), yet the total reduction of influenza infections in the German population is only 554,000 infections per year if pre-school children are targeted whereas it is 746,000 if children of all ages are targeted. This indicates that including older children and juveniles in a vaccination programme may be particularly effective in preventing infections in the community. As high-risk conditions occur among all children at a frequency of less than 10%, restricting pediatric vaccinations on this subgroup can have only a modest effect on the overall transmission. Extending the present risk-based vaccination practice in Germany by increasing the coverage rate of all at-risk children from the current value of about 7.5% even to 90% would only prevent 376,000 infections annually in the total German population.

Due to the stochasticity of our simulations, we obtain widely different results for the same simulation year even without changing any simulation parameters. In order to compensate for this variability, we report median results when we compare our simulation results with field observations. Results from randomized controlled trials (RCT) or epidemiologic field studies can only cover a small time period which does not capture this kind of variability; the validity of their results is frequently expressed by 95% confidence intervals, but these intervals cannot predict what would have happened in a different time period. This fundamental difference has to be kept in mind when comparing stochastic simulation results with field observations.

Using representative data from Germany 2008–10, Sauerbrei et al.^37^ examined how sero-prevalence against any of the Influenza A strains or against any of the Influenza B lineages increased over age. For Influenza A, their age-dependent sero-prevalence values are slightly higher than those obtained by our simulations (see Figure A2a). A reason for this may be that most of the serum samples tested by Sauerbrei et al.^37^ were taken in 2010, shortly after the first A(H1N1) pandemic wave in Germany, when an unusually high number of children got infected with (pandemic) Influenza A virus.^38^ For Influenza B, age-dependant sero-prevalence values are lower than those obtained by our simulations (Figure A2b), however for the combined age-dependent sero-prevalence for any influenza, there is an almost perfect match with our results (Figure A2c). When comparing to representative sero-prevalence data from the Netherlands,^39^ our simulation, again, slightly under-estimates sero-prevalence for Influenza A (Figure A2a), while closely matching sero-prevalence for Influenza B (Figure A2b). As for Germany, the combined age-dependent sero-prevalence for any influenza from the Netherlands matches very well with the results of our simulations (Figure A2c). There are several limitations when comparing our simulation results to data from the Netherlands: (1) simulated years (2008/09 to 2010/11) do not match the observation period from the study (2006/07); (2) demography and the matrix of contact^17^ as well as vaccination coverage in the Netherlands^40^ differs from Germany. Overall, our median seroprevalence age-profiles for any influenza infection are well in line with published data from Germany and the Netherlands.

The age-specific transmission pattern was derived from an output value randomly picked and not over multiple simulations which is a limitation in our model. In addition, a general limitation may be that the network which connects individuals in our simulations is based on the German POLYMOD study from 2005/2006^20^ and that, theoretically, contact behavior may have changed since. Furthermore, the POLYMOD study reported social contact behavior from healthy individuals which may differ from individuals symptomatically infected with influenza.

In summary, our modeling results strongly support, that the introduction of yearly routine vaccination against influenza in children and juveniles is an effective measure to substantially lower the disease burden both in the target vaccine group and in the general population.
