# Supplementary material for: Assessing direct and indirect effects of pediatric influenza vaccination in Germany by individual-based simulations
Source: Hum Vaccin Immunother. 2019 Dec 6;16(4):836–45. doi: 10.1080/21645515.2019.1682843 (PMC7227695; doi:10.1080/21645515.2019.1682843)
Supplement: Supplemental Material [file khvi-16-04-1682843-s001.docx]

**Online supporting material**

Table A1. List of model parameters taken from [17]

| Parameter | Value | Reference |
| --- | --- | --- |
| Average transmission probability per day per contact | 0.0305 | calculated from [28] |
| Maximum seasonal transmission factor | 1.43 | [25] |
| Day of maximum seasonal transmission | Dec. 21st | [25] |
| Duration of the latent period | 2 days | [32] |
| External infection probability | 0.001/year | assumed |
| Duration of the infectious period  - children (age 0–17 years)  - adults (age 18 years and above) | 4 days  2 days | [32] |
| Duration of maternal protection | 2 - 4 months | [33;34] |
| Immunity loss rate after infection | 1 / (9.13 years) | calculated from [25], see [17] |
| Average circulation time per drift variant  - A(H1N1), B/Vic, B/Yam  - A(H3N2) | 7.0 years  3.5 years | calculated from [35;36], see [17] |
| Revaccination preference factor | 4.25 | [16] |
| Probability of mismatched vaccine design when a new drift variant occurs | 40% | calculated from [17-19;35;36], see [5] |
| Vaccine efficacy against RT-PCR confirmed symptomatic influenza  - 0–2 years of age  - 3–8 years of age  - 9–15 years of age  - 16–64 years of age  - 65 years of age or older | 49.8%  55.4%  69.0%  63.0%  58.0% | [6]  [37]  [3]  [5]  [4] |
| Cross protection after infection  - percentage of individuals who are immunized against a B lineage when they are infected (or boosted) with the other B lineage (lineage cross protection)  - percentage of individuals who were immune against the previous drift variant, who are still protected against the new one (drift cross protection) | 60%  60% | calculated from [38], see [17] |
| Cross protection after vaccination  - vaccine efficacy multiplication factor for B lineage not contained in TIV (lineage cross protection)  - vaccine efficacy multiplication factor for vaccinations with drift mismatch (drift cross protection) | 0.6  0.6 | calculated from [38], see [17] |
| Immunity loss after vaccination | 100% at the end of each simulation year | assumed |
| Percentage of the population with elevated risk  - newborn individuals  - age 0 to 15 years  - age 16 to 59 years  - age 60 years and above | 3.0%  6.0%  14.2%  47.1% | [39] |
| Percentage immune before initialization of simulations begins | 35% | assumed |

Table A2. Baseline vaccination coverage.

| Age group (years) | Annual vaccination coverage | Reference |
| --- | --- | --- |
| 0-17 | Average over all children 0.5-17 years: 4.1%.  Distributed proportionally, assuming that the vaccination coverage of at-risk children was twice as high as that of no risk children:  0.5-15 years, low risk: 3.87%, high risk: 7.74%;  16-17 years, low risk: 3.59%, high risk: 7.18%. | [40] |
| 18-39 | Average of all individuals: 13.6%.  Distributed proportionally, assuming that the vaccination coverage of at-risk individuals was twice as high as that of no risk individuals:  low risk: 11.91%, high risk: 23.82%. | [39] |
| 40-59 | Average of all individuals: 22.2%.  Distributed proportionally, assuming that the vaccination coverage of at-risk individuals was twice as high as that of no risk individuals:  low risk: 19.44%, high risk: 38.88% | [39] |
| 60+ | Coverage until 2009: 47.7%.  Linear decline thereafter until 2015/16 to 33.2%.  Coverage kept constant after 2016 at 33.2%. | [11]  [41] (assumed) |

Table A3. Direct and indirect effects of vaccinating a percentage of children from 6 months to 17 years of age with QIV in a population of 100,000 individuals with German demography (1,000 pairs of simulations for each coverage; evaluation period 20 years). The results show the average number of annually prevented infections and the percentage or reduction in the respective age group relative to the numbers of infections which occur in branch 1 (QIV vaccination at baseline level), which were set to 100%.

| Coverage | Children | | | Adults | | | Elderly | | All | | |
| --- | --- | --- | --- | --- | --- | --- | --- | --- | --- | --- | --- |
|  | N | | (%;  Q1-Q3) | N | | (%;  Q1-Q3) | N | (%;  Q1-Q3) | N | | (%;  Q1-Q3) |
| 10% | 218 | (6.32%; 4.4-8.3) | | 204 | (2.90%; 0.7-5.0) | | 95 | (3.11%; 0.5-5.7) | 517 | (3.83%; 1.7-6.0) | |
| 15% | 397 | (11.47%; 9.6-13.4) | | 375 | (5.29%; 3.3-7.5) | | 173 | (5.63%; 3.2-8.3) | 945 | (6.95%; 5.0-9.2) | |
| 20% | 574 | (16.75%; 14.7-18.8) | | 560 | (8.04%; 5.7-10.3) | | 258 | (8.58%; 5.9-11.3) | 1392 | (10.40%; 8.0-12.8) | |
| 25% | 744 | (21.58%; 19.8-23.7) | | 738 | (10.52%; 8.3-12.7) | | 338 | (11.16%; 8.6-13.8) | 1820 | (13.50%; 11.3-15.8) | |
| 30% | 913 | (26.46%; 24.3-28.6) | | 930 | (13.26%; 11.0-15.6) | | 422 | (13.94%; 11.3-16.6) | 2265 | (16.80%; 14.6-19.1) | |
| 35% | 1063 | (30.86%; 28.6-33.0) | | 1098 | (15.69%; 13.3-18.2) | | 495 | (16.41%; 13.8-19.2) | 2656 | (19.74%; 17.3-22.2) | |
| 40% | 1216 | (35.20%; 32.9-37.4) | | 1277 | (18.18%; 15.7-20.7) | | 574 | (18.94%; 16.2-21.8) | 3066 | (22.71%; 20.3-25.2) | |
| 45% | 1349 | (39.37%; 36.7-41.8) | | 1438 | (20.69%; 17.9-23.3) | | 643 | (21.46%; 18.4-24.3) | 3429 | (25.65%; 22.9-28.2) | |
| 50% | 1488 | (43.16%; 40.6-45.6) | | 1619 | (23.12%; 20.5-25.5) | | 723 | (23.91%; 21.0-26.6) | 3830 | (28.43%; 25.7-30.9) | |
| 55% | 1608 | (46.80%; 44.2-49.6) | | 1783 | (25.56%; 22.7-28.4) | | 792 | (26.33%; 23.3-29.5) | 4183 | (31.18%; 28.3-34.1) | |
| 60% | 1732 | (50.48%; 47.8-53.0) | | 1961 | (28.19%; 25.4-30.8) | | 868 | (28.92%; 25.8-32.0) | 4561 | (34.07%; 31.4-36.7) | |

Q1-Q3, 25^th^ and 75^th^ percentile; QIV, quadrivalent inactivated influenza vaccine.

Table A4. Direct and indirect effects of vaccinating a percentage of children from 6 months to 4 years of age with QIV in a population of 100,000 individuals with German demography (1,000 pairs of simulations for each coverage; evaluation period 20 years). The results show the average number of annually prevented infections and the percentage or reduction in the respective age group relative to the numbers of infections which occur in branch 1 (QIV vaccination at baseline level), which were set to 100%.

| Coverage | Children | | Adults | | Elderly | | All | |
| --- | --- | --- | --- | --- | --- | --- | --- | --- |
|  | N | (%;  Q1-Q3) | N | (%;  Q1-Q3) | N | (%;  Q1-Q3) | N | (%;  Q1-Q3) |
| 10% | 44 | (1.24%; -0.8-3.3) | 48 | (0.61%; -1.6-2.9) | 21 | (0.62%; -2.2-3.3) | 114 | (0.78%;  -1.4-3.0) |
| 15% | 80 | (2.25%; 0.3-4.3) | 77 | (1.03%; -1.1-3.3) | 32 | (0.97%; -1.6-3.7) | 188 | (1.33%; -1.8-3.6) |
| 20% | 120 | (3.48%; 1.7-5.3) | 130 | (1.82%; -0.3-3.9) | 56 | (1.79%; -0.8-4.4) | 306 | (2.24%; 0.1-4.3) |
| 25% | 150 | (4.33%; 2.3-6.4) | 155 | (2.18%; 8.3-12.7) | 69 | (2.20%; 8.6-13.8) | 374 | (2.74%; 11.3-15.8) |
| 30% | 182 | (5.25%; 3.3-7.2) | 182 | (2.55%; 0.3-4.8) | 77 | (2.49%; -0.2-5.2) | 442 | (3.23%; 1.0-5.4) |
| 35% | 218 | (6.29%; 4.3-8.3) | 222 | (3.14%; 0.8-5.5) | 94 | (3.07%; 0.4-5.7) | 534 | (3.93%; 1.6-6.2) |
| 40% | 251 | (7.23%; 5.4-9.1) | 261 | (3.67%; 1.5-5.8) | 112 | (3.63%; 1.1-6.1) | 624 | (4.58%; 2.4-6.7) |
| 45% | 282 | (8.21%; 6.3-10.1) | 295 | (4.21%; 2.2-6.4) | 125 | (4.13%; 1.6-6.7) | 702 | (5.22%; 3.1-7.4) |
| 50% | 311 | (8.98%; 6.9-10.9) | 325 | (4.59%; 2.3-6.8) | 140 | (4.55%; 1.9-7.1) | 776 | (5.71%; 3.5-7.9) |
| 55% | 340 | (9.87%; 8.1-11.7) | 356 | (5.08%; 2.9-7.3) | 152 | (5.00%; 2.3-7.4) | 847 | (6.29%; 4.2-8.4) |
| 60% | 372 | (10.79%; 8.9-12.7) | 394 | (5.59%; 3.4-7.6) | 169 | (5.53%; 3.1-8.0) | 934 | (6.91%; 4.8-8.9) |

Q1-Q3, 25^th^ and 75^th^ percentile; QIV, quadrivalent inactivated influenza vaccine.

Table A5. Univariate sensitivity analyses of the direct and indirect effects of vaccinating 40% of children from 6 months to 17 years of age with QIV in a population of 100,000 individuals with German demography (averages of 1,000 pairs of simulations for each parameter setting; evaluation period 20 years). The results show the average number of annually prevented infections and the percentage or reduction in the respective age group relative to the numbers of infections which occur in branch 1 (QIV vaccination at baseline level), which were set to 100%.

| Variation | Children | | Adults | | Elderly | | All | |
| --- | --- | --- | --- | --- | --- | --- | --- | --- |
|  | N | (%;  Q1-Q3) | N | (%;  Q1-Q3) | N | (%;  Q1-Q3) | N | (%;  Q1-Q3) |
| Low Immunity Duration | 1587 | (34.14%; 32.2-36.0) | 1706 | (16.91%; 14.9-18.9) | 778 | (18.13%; 15.9-20.3) | 4071 | (21.40%; 19.4-23.5) |
| Baseline Immunity Duration | 1216 | (35.20%; 32.9-37.4) | 1277 | (18.18%; 15.7-20.7) | 574 | (18.94%; 16.2-21.8) | 3066 | (22.71%; 20.3-25.2) |
| High Immunity Duration | 1045 | (36.47%; 33.5-39.4) | 1077 | (19.67%; 16.7-22.8) | 475 | (20.04%; 16.7-23.5) | 2597 | (24.26%; 21.2-27.4) |
| Low Infection Probability | 1162 | (40.80%; 38.4-43.6) | 1400 | (24.64%; 21.5-27.5) | 584 | (24.82%; 21.5-28.1) | 3146 | (28.92%; 26.0-31.7) |
| Baseline Infection Probability | 1216 | (35.20%; 32.9-37.4) | 1277 | (18.18%; 15.7-20.7) | 574 | (18.94%; 16.2-21.8) | 3066 | (22.71%; 20.3-25.2) |
| High Infection Probability | 1237 | (31.35%; 29.3-33.4) | 1136 | (13.93%; 11.9-15.9) | 549 | (15.04%; 12.7-17.5) | 2922 | (18.56%; 16.5-20.6) |
| Low Vaccine Efficacy | 1012 | (29.03%; 26.9-31.2) | 1041 | (14.78%; 12.5-17.1) | 468 | (15.37%; 12.8-18.1) | 2521 | (18.58%; 6.3-21.0) |
| Baseline Vaccine Efficacy | 1216 | (35.20%; 32.9-37.4) | 1277 | (18.18%; 15.7-20.7) | 574 | (18.94%; 16.2-21.8) | 3066 | (22.71%; 20.3-25.2) |
| High Vaccine Efficacy | 1348 | (39.27%; 36.9-41.7) | 1445 | (20.61%; 17.9-23.3) | 649 | (21.47%; 18.5-24.4) | 3442 | (25.57%; 22.9-28.2) |

Q1-Q3, 25^th^ and 75^th^ percentile; QIV, quadrivalent inactivated influenza vaccine.

Table A6. Univariate sensitivity analyses of the direct and indirect effects of vaccinating 40% of children from 6 months to 4 years of age with QIV in a population of 100,000 individuals with German demography (averages of 1,000 pairs of simulations for each parameter setting; evaluation period 20 years). The results show the average number of annually prevented infections and the percentage or reduction in the respective age group relative to the numbers of infections which occur in branch 1 (QIV vaccination at baseline level), which were set to 100%.

| Variation | Children | | | Adults | | | Elderly | | | All | | |
| --- | --- | --- | --- | --- | --- | --- | --- | --- | --- | --- | --- | --- |
|  | N | | (%;  Q1-Q3) | N | | (%;  Q1-Q3) | N | | (%;  Q1-Q3) | N | | (%;  Q1-Q3) |
| Low Immunity Duration | 329 | (7.05%; 5.6-8.6) | | 358 | (3.52%; 1.9-5.2) | | 156 | (3.59%; 1.7-5.5) | | 843 | (4.40%; 2.8-6.1) | |
| Baseline Immunity Duration | 251 | (7.23%; 5.4-9.1) | | 261 | (3.67%; 1.5-5.8) | | 112 | (3.63%; 1.1-6.1) | | 624 | (4.58%; 2.4-6.7) | |
| High Immunity Duration | 207 | (7.18%; 4.9-9.7) | | 209 | (3.74%; 1.0-6.4) | | 85 | (3.50%; 0.7-6.8) | | 501 | (4.61%; 2.0-7.4) | |
| Low Infection Probability | 222 | (7.75%; 5.2-10.3) | | 268 | (4.63%; 1.8-7.3) | | 108 | (4.48%; 1.1-7.8) | | 597 | (5.42%; 2.6-8.2) | |
| Baseline Infection Probability | 251 | (7.23%; 5.4-9.1) | | 261 | (3.67%; 1.5-5.8) | | 112 | (3.63%; 1.1-6.1) | | 624 | (4.58%; 2.4-6.7) | |
| High Infection Probability | 273 | (6.89%; 5.3-8.4) | | 251 | (3.04%; 1.4-4.7) | | 112 | (3.00%; 1.0-5.0) | | 636 | (4.00%; 2.4-5.6) | |
| Low Vaccine Efficacy | 207 | (5.90%; 4.0-7.9) | | 222 | (3.11%; 0.9-5.4) | | 97 | (3.13%; 0.5-5.8) | | 526 | (3.84%; 1.6-6.1) | |
| Baseline Vaccine Efficacy | 251 | (7.23%; 5.4-9.1) | | 261 | (3.67%; 1.5-5.8) | | 112 | (3.63%; 1.1-6.1) | | 624 | (4.58%; 2.4-6.7) | |
| High Vaccine Efficacy | 284 | (8.24%; 6.4-10.1) | | 297 | (4.21%; 2.0-6.4) | | 127 | (4.13%; 1.4-6.8) | | 707 | (5.22%; 3.0-7.4) | |

Q1-Q3, 25^th^ and 75^th^ percentile; QIV, quadrivalent inactivated influenza vaccine.

Figure A1. Univariate sensitivity analyses for direct and indirect effects of vaccinating 40% of children from 6 months to 4 years of age with QIV in a population of 100,000 individuals with German demography (annually prevented infections; black: children 0-17 years, dark grey: young adults 18-59 years, light grey: elderly 60+ years; averages of 1,000 pairs of simulations for each parameter setting; evaluation period 20 years).


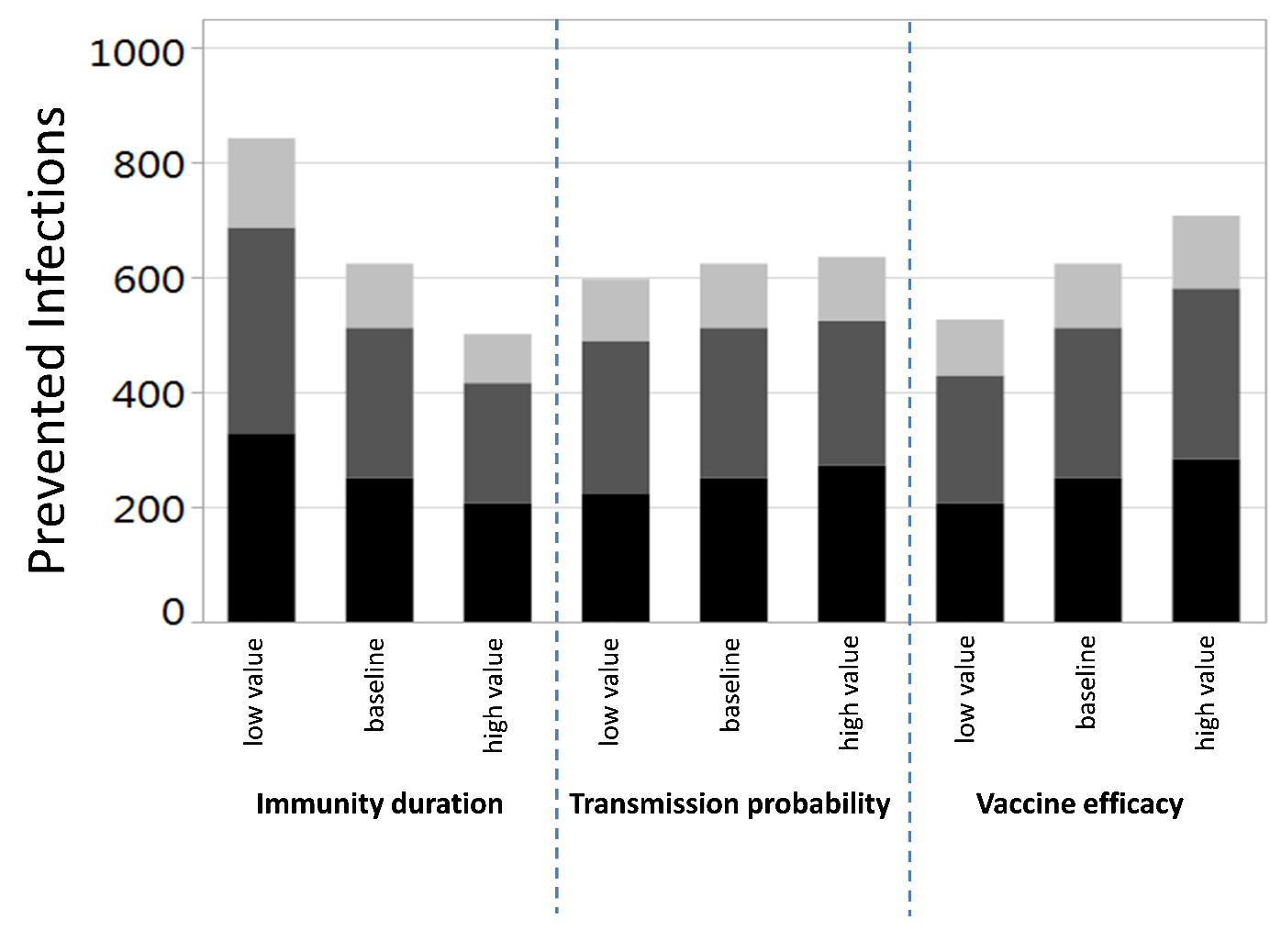


QIV, quadrivalent inactivated influenza vaccine.

**Figure A2**. Infection-derived seroprevalence against (a) any one of the two Influenza A strains, (b) any one of the two Influenza B lineages, and (c) against any influenza infection for the seasons 2008/09 to 2010/11. Results were obtained from 3,000 simulations with 100,000 individuals each. Full lines show the age-dependent median sero-prevalence; dotted lines denote 95% range of the simulation results. Grey squares show the results reported by Bodewes et al. [38]; black circles show the results reported by Sauerbrei et al. [36] whereby their grouped 2 years age intervals are displayed as two circles.

(a)


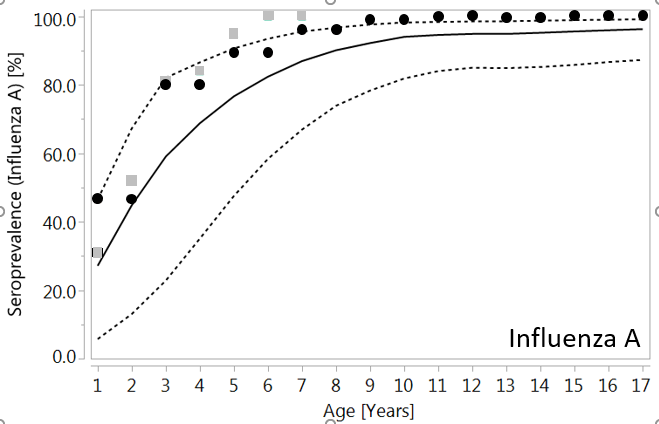


**(b)**


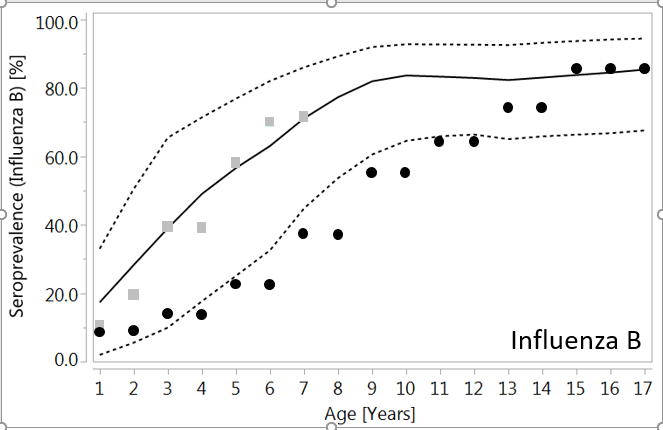


**(c)**


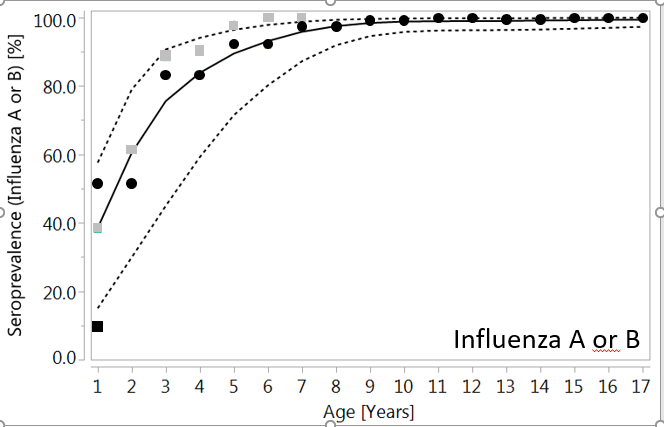


**Basic reproduction number R_0_**

In order to translate the calibrated infection probability per contact per day into a more common measure of transmissibility, we have also calculated a basic reproduction number for the simulations. To do so, we re-interpreted the contact network as a sparsely populated 100,000 x 100,000 contact matrix which mostly consists of zeros and a few ones (denoting individuals who are in contact with each other). Multiplying this matrix with the time-specific infection probability per contact per day and with the age-specific duration of infectiousness provided us with the Next Generation Matrix for a fully susceptible population. The largest eigenvalue of this matrix can then be considered as the basic reproduction number R_0_. As the infection probability is assumed to change periodically over the year, the maximum eigenvalue depends on the time of the year. As the contact structure also depends on the demographic composition of the population, the largest eigenvalue furthermore depends on the demography of Germany which changes during the simulation. Calculating the all-year average of the largest eigenvalue leads to an estimate of 1,38 at the beginning of the evaluation period (i.e. in 2018). Due to the ageing population, this value drops to 1.30 until the end of the evaluation period (i.e. the year 2037).

Drift variants, vaccine mismatch, and cross protection

(text reproduced from [17])

Smith et al. [35] constructed an antigenic map of Influenza A(H3N3) data from 35 years of surveillance (1968 to 2003) and found 11 antigenically distant clusters; the average antigenic distance between the centers of consecutive clusters was 4.5 units (SD 1.3). As influenza vaccines are updated when there is an antigenic difference of at least 2.0, these clusters can be regarded as representing genetically relevant drift variants. As ten new drift variants occurred in the time period of 35 years, we get an average duration of D = 3.5 years for new A(H3N2) drift variants (95% CI from 2.2 to 6.8). No direct estimates for the duration between consecutive Influenza B drift variants are available, but as they change at a rate which is about two to three times lower than this [3], we assume the average sojourn time of Influenza B drift variants to be 7 years. Judging by the frequency of changes in vaccine composition from 1987 to 2000, the variability of A(H1N1) may be similar to that of Influenza B [3]; we, therefore, use the same average duration of 7 years for Influenza A(H1N1) drift variants. For each one of the two A subtypes and the two B lineages, an independent random number r is calculated at the beginning of each simulation year to determine whether the circulating variant is replaced by a new drift variant. If r < 1/D, a new drift variant is introduced.

The possibility of a vaccine design mismatch is only considered for years in which new drift variants appear. Of course, TIV vaccination can only be affected by an Influenza B mismatch, if the mismatched B lineage is actually contained in the vaccine. For the last 18 years, four mismatch events have been reported (A(H3N2) in 2003 and 2004, and A(H1N1) in 2006 and 2009 [40]). For this time period, we calculate that 18/3.5 = 5.14 new drift variants for A(H3N2) and 18/7 = 2.57 new drift variants for A(H1N1) appeared. For the B lineage which was included in TIV during the 18 years, we also calculate 18/7 = 2.57 new drift variants. As we expect altogether 10.28 drift events and observed 4 mismatch events, we calculate a mismatch probability of 40% per drift. We use this probability for all four influenza variants, including the two B lineages for which no vaccine design mismatch has been reported.

The immunity shared between a previously circulating variant and a new variant is not well known. We infer information on this shared immunity from vaccination studies summarized by Jefferson et al. [39]: well-matched TIV vaccination protects on average 73% of healthy adults, whereas mismatched TIV vaccination protects only 44%. We take the ratio of 44/73 = 60% also as indicator for the shared immunity between subsequent drift variants. 60% of the individuals who were immune against the previously circulating variant are also immune against the new drift variant, whereas the remaining 40% are susceptible to the new drift variant. For the sake of simplicity, we replace variants by new drift variants at the beginning of a simulation year (i.e. on July 1st). We do not allow co-circulation of the old and the new variant in the simulation, and we assume that a previously circulating variant will never be introduced again.

We furthermore use this estimate of 60% for shared immunity between the two B lineages: if an individual is infected with one B lineage, an additional immunization (or booster event) with respect to the other lineage takes place with a probability of 60%. Likewise, TIV vaccination can cause immunity (or booster events) against the B lineage which is not contained in the vaccine; the age-dependent vaccine efficacy is multiplied by 0.6 to obtain the efficacy against the missing lineage. We restrict this cross-immunizing vaccination effect to TIV because there is no convincing evidence that increasing the antigen amount enhances the efficacy of influenza vaccines [57-59]. Tricco et. al. [10] report for vaccinees from 18 to 49 years of age that the vaccine efficacy against Influenza B is 77% if the lineage is contained in TIV and 52% if it is not contained in TIV, respectively. The ratio 52/77 = 0.675 indicates a B lineage cross protection of 67.5%. Langley et al. examine in a vaccination study with children what percentages seroconvert with respect to the B lineage which is contained and which is not contained in TIV. As they use two different TIV vaccines which either contain B/Vic or B/Yam, they obtain four different sero-conversion rates with respect to the Influenza B lineages [59] (summarized in Table 3). We have developed a maximum likelihood model based on the assumption that either one of the two TIV vaccines causes a direct sero-conversion S with respect to the B lineage contained in the vaccine and an indirect effect S x with respect to the lineage not contained in the vaccine. The joint likelihood L of the observations is then given by

__, omitting the constant factors, this simplifies to __.

The maximum likelihood estimates are S = 72.5% (95% confidence interval CI: 70.3-74.5, based on the profile likelihood) and x = 49.1% (CI: 45.7-52.5), respectively, i.e. the sero-conversion rate of the B lineage not contained in TIV is about half as high as that of the lineage which is contained in the vaccine. Having obtained three indicators for the amount of B lineage cross protection (49.1% Langley, 60% Jefferson and 67.5% Tricco, respectively), we have decided to use the value 0.6 as the baseline value in our simulation studies.

**Table 3**. Sero-conversion rates as reported by Langley et al. [59], defined as the proportion of vaccinees with a pre-vaccination titer <1:10 and a post-vaccination titer ≥1:40, or a pre-vaccination titer ≥1:10 and at least a 4-fold increase in post-vaccination titer.

| TIV vaccination group | Seroconversion rate | |
| --- | --- | --- |
|  | with respect to B Yamagata | with respect to B Victoria |
| TIV-Vic (n = 870) | 41.3% (n = 359) | 71.5% (n = 622) |
| TIV-Yam (n = 877) | 73.4% (n = 644) | 29.9% (n = 262) |

TIV-Vic, trivalent influenza vaccine with Victoria lineage B strain; TIV-Yam, trivalent influenza vaccine with Yamagata lineage B strain. The numbers of sero-converters (n) were calculated from the percentages.
